# Case of Peculiar Nervous Excitement

**Published:** 1826-01

**Authors:** J. Vale Asbury

**Affiliations:** Enfield.


**Art. IV.**—*Case of peculiar Nervous Excitement.* By J. Vale Asbury, Esq. Enfield.

On perusing a late Number of the London Medical and Physical Journal, two "cases of hydrophobia, not arising from the bite of a rabid animal, by — Whymper, m.d. surgeon-major 2d regiment of Guards," attracted particular attention, and forcibly called to my recollection the following case, which I extract from notes taken on visiting the patient. I present it without comment, and leave the medical world to make such inductions as the occurrence of similar cases may ultimately offer.

June 23d, 1822.—Smith, a labourer, ætat. twenty-three, of small stature, general health good, (had been employed in the hay-fields during a fortnight of very hot weather,) was taken in a fit at the time a barber was cutting his hair. It began by tremors, anxiety, a sense of weight and oppression at the chest, with apparent suffocation and difficulty of swallowing; the countenance exhibiting general symptoms of alarm, and the whole body much agitated. In about three-quarters of an hour, these symptoms left him spontaneously; he became tranquil in body and mind, and then complained only of slight pain in the head, with general debility. The tongue white; pulse sixty-five, small and soft.—Ordered four grains of calomel, with a strong cathartic.

24th.—He had no return of the symptoms, but complained of giddiness and pain in the head. Did not see him this day.

25th.—Let blood, to eight ounces: the pulse previously about sixty, and contracted; after bleeding, it was somewhat more expanded. He walked two miles afterward; and, when at home, the arm bled again, and he was speedily taken in another fit, which presented the character above stated, but with greater sense of suffocation and difficulty in swallowing. The idea of being compelled to swallow any liquid produced great agony. The paroxysms continued from three to five minutes, and then the system became tranquil for about the same period of time. These paroxysms, with their intervals of collapse, continued an hour,—from four to five o'clock of this day. I saw him about half after five, and remained in his room three-quarters of an hour: at this time he had no pain any where, but complained of slight giddiness in the head, and a weight above the sternum, with general languor; in other respects, he called himself well. He swallowed an antispasmodic draught, in divided portions, tolerably well; but the last portion occasioned choking, which soon went off again, without any further inconvenience; and he had no return of fits till twelve o'clock at night. During this time he appeared watchful, restless, and had a peculiar degree of anxiety depicted in his countenance. From twelve to one o'clock at night, the fits continued in the form stated from four to five of this day; when they again left him, and he became tranquil, and had some sleep.

26th.—He ate some light food, and drank freely. He complained of great weakness, and appeared restless as well as watchful. About four o'clock, he lost his speech for upwards of an hour. The evening of this day he complained of hunger, and ate a mutton-chop. He had some sleep in the night, but was generally restless.

27th.—In the morning, great restlessness and watching; in the evening, frequent fits of laughter, and from eleven o'clock continued laughing violently for three-quarters of an hour.

28th.—He was removed to the Middlesex Hospital, and there he immediately fell into a state of stupor, which continued for some days. By the employment of purgative medicines he recovered, excepting from debility, and left the hospital.

He continues to follow his employment as a labourer, and has had no return of his disorder. Purgatives, with antispasmodics, formed the general plan of treatment, previously to his admission into the hospital.

